# Short-term changes in the anterior segment and retina after small incision lenticule extraction

**DOI:** 10.1186/s12886-020-01668-7

**Published:** 2020-10-07

**Authors:** Yanwei Chen, Huaping Liao, Yue Sun, Xi Shen

**Affiliations:** Department of Ophthalmology, Ruijin Hospital Affiliated Shanghai Jiao Tong University, School of Medicine, Shanghai, China

**Keywords:** Small incision lenticule extraction, Anterior segment, Retina, Optical coherence tomography angiography (OCTA)

## Abstract

**Background:**

To analyse short-term changes in the anterior segment and retina after small incision lenticule extraction (SMILE).

**Methods:**

Patients with myopia scheduled for SMILE were recruited from Ruijin Hospital, Shanghai, China. Basic patient information such as age, sex, and refractive errors was recorded. Ocular measurements were taken before surgery, and 1 day and 1 week after surgery; they included axial length (AL), central corneal thickness (CCT), anterior chamber depth (ACD), lens thickness (LT), white to white (WTW), pupil diameter (PD), macular thickness (MT), ganglion cell layer thickness (GCL), retinal nerve fiber layer thickness (RNFL), choroidal thickness (CT), macular vessel density, and optic disc vessel density.

**Results:**

Sixty-one eyes of 31 patients were selected for this study. AL, CCT, ACD, and postoperative PD were significantly reduced (*p* < 0.05), while LT was thickened after surgery (p < 0.05). MT at the fovea decreased 1 day and 1 week after surgery (p < 0.05). GCL showed no significant changes after surgery. RNFL was unchanged 1 day after surgery, but the inferior sector was thickened 1 week after surgery. CT was thicker at the fovea 1 day after surgery and 1.0 mm from the fovea in the nasal sector 1 week after surgery. Macular vessel density was significantly decreased 1 day after surgery and most recovered in 1 week. Optic disc vessel density decreased at the peripapillary part 1 day after surgery and recovered after 1 week. ΔACD and ΔLT showed no significant correlation 1 day after surgery. ΔACD was negatively correlated with ΔLT and sphere 1 week after surgery (*r* = − 0.847, *p* < 0.000; r = − 0.398, *p* = 0.002). ΔLT was positively correlated with the sphere 1 week after surgery (r = 0.256, *p* = 0.048).

**Conclusion:**

The anterior segment was the most affected, while the retina also underwent changes with regard to MT, RNFL, CT, macular vessel density, and peripapillary vessel density.

## Background

Laser refractive surgery has been developing for more than 30 years since Dr. Steven Trokel and his colleagues first reported photorefractive keratectomy (PRK) in 1983 [1]. Subsequently, laser in situ keratomileusis (LASIK), and laser subepithelial keratomileusis (LASEK) developed. About 10 years ago, small-incision lenticule extraction (SMILE) gradually came up and now has become the most popular surgery for refraction correction for its effectiveness, stability, and safety [2, 3].

As myopia patients grow, the effects of refractive surgeries also attract doctors’ concerns. LASIK and SMILE are so similar that researchers have compared them in different ways. Changes in LASIK have been studied extensively. Cornea biomechanics decrease after both LASIK and SMILE [4, 5]. Posterior corneal elevation (PCE) and anterior chamber depth (ACD) are also reduced [6, 7], but few studies have investigated changes in the retina.

Optical coherence tomography angiography (OCTA) has predominated now-a-days for its deep scan and retinal vascular quantitative analysis. Previous studies have shown that retinal microvascularity decreases in myopia patients [8, 9]. Prior studies have suggested that suction during surgery is a crucial factor responsible for all ocular changes [10, 11]. In this study, we measured both the anterior and posterior parameter outcomes after SMILE.

## Methods

### Participants

This was a prospective observational study. The design and procedure of this study adhered to the principles of the Declaration of Helsinki. The Institutional Review Board of Ruijin Hospital authorised this study. Patients who were willing to undergo SMILE at Ruijin Hospital from August 2019 to December 2019 were enrolled. Written informed consent was obtained from each subject.

The inclusion criteria were as follows: age > 18 years, corrected distance visual acuity no less than 20/20, without any ophthalmologic or systematic disease, stable myopia for more than two years, and calculated residual stromal bed > 250 μm.

### Measurement of clinical examination

All participants underwent a complete ophthalmologic examination before and 1 day, 1 week after the surgery, including visual acuity assessment, intraocular pressure (IOP), and refraction. Axial length (AL), central corneal thickness (CCT), anterior chamber depth (ACD), lens thickness (LT), white to white (WTW), and pupil diameter (PD) were measured using Lenstar (LS 900) and corneal tomography captured with Patencam (Oculus, Wetzlar, Germany). Changes of ACD and LT were recorded as ΔACD (ACD after surgery - ACD before surgery) and ΔLT (LT after surgery - LT before surgery).

OCT scans were captured with Cirrus HD OCT 5000(Carl Zeiss Meditec) software version 9.5.2, and analysed with a software version of 10.0.0. Five types of protocols were used to obtain images. Macular thickness (MT) and ganglion cell layer thickness (GCL) were obtained with a Macular Cube 512 × 128. Retinal fiber layer thickness (RNFL) was obtained with Optic Disc Cube 200 × 200, and choroidal thickness (CT) was obtained with Angiography 3 × 3 mm enhanced depth imaging (EDI) mode, and the superficial vascular density of the macular and optic disk was obtained with angiography 6 × 6 mm.

**Fig. 1:**
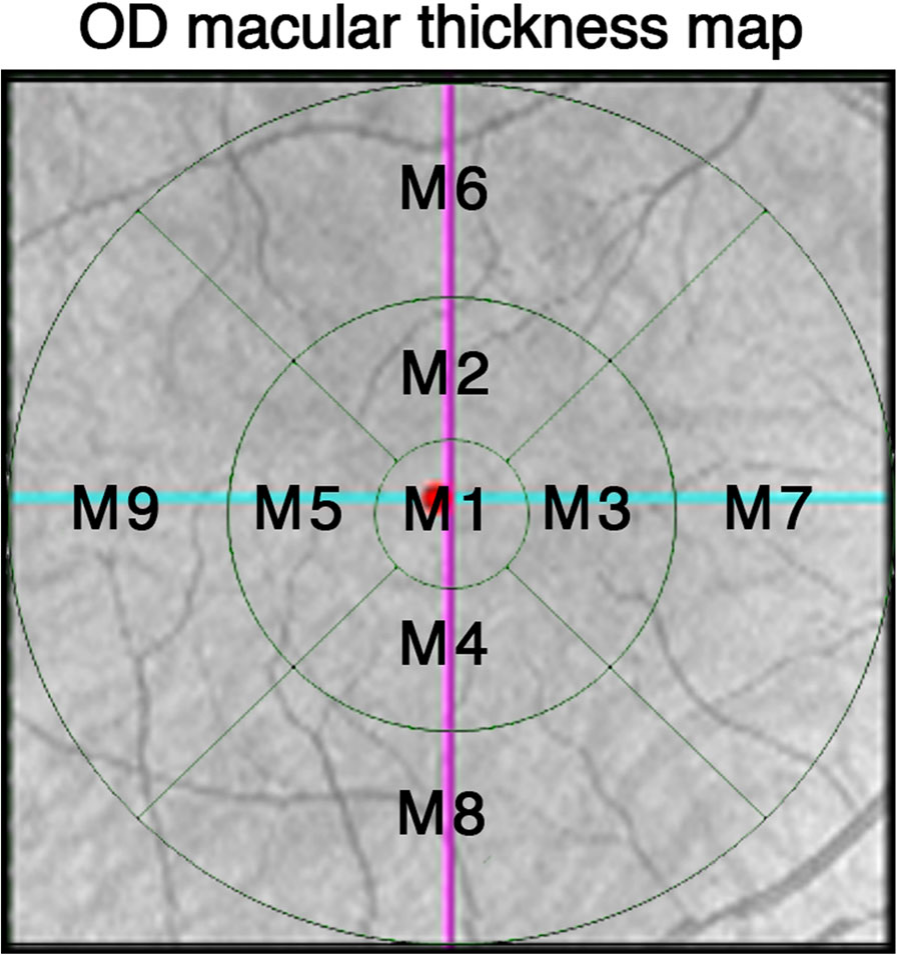
Macular thickness map of the right eyes based on OCT

**Fig. 2:**
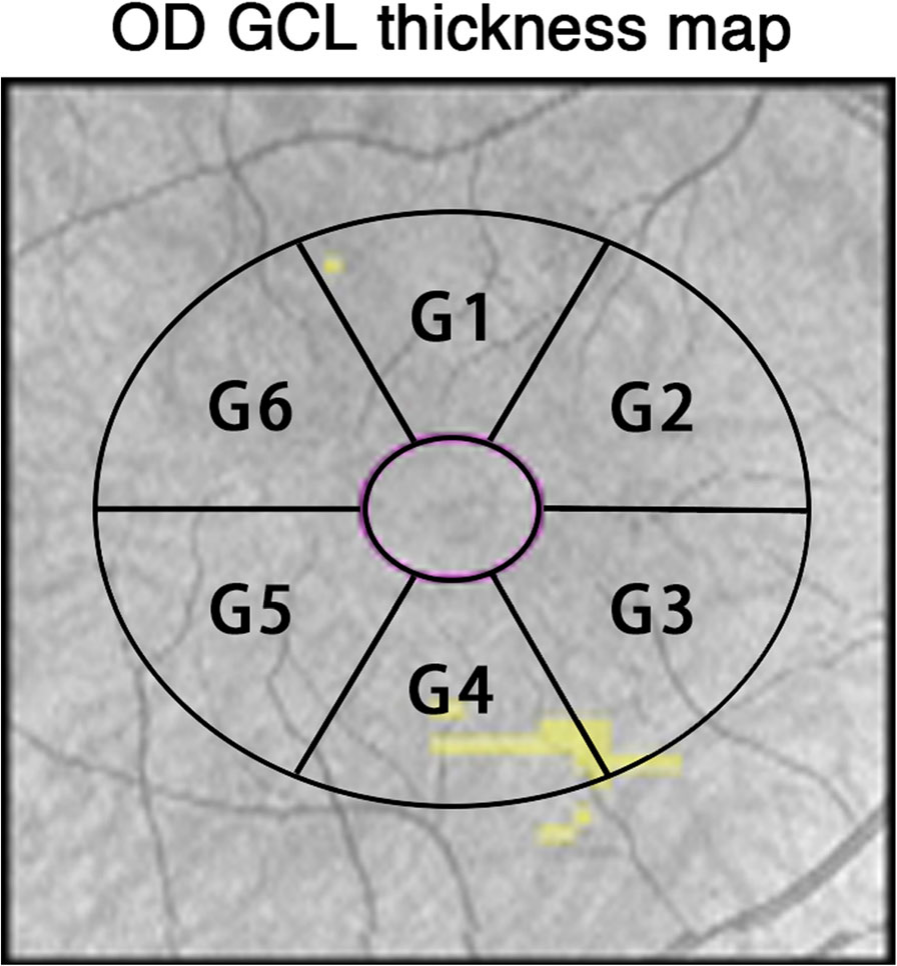
Ganglion cell layer thickness map of the right eyes based on OCT

**Fig. 3:**
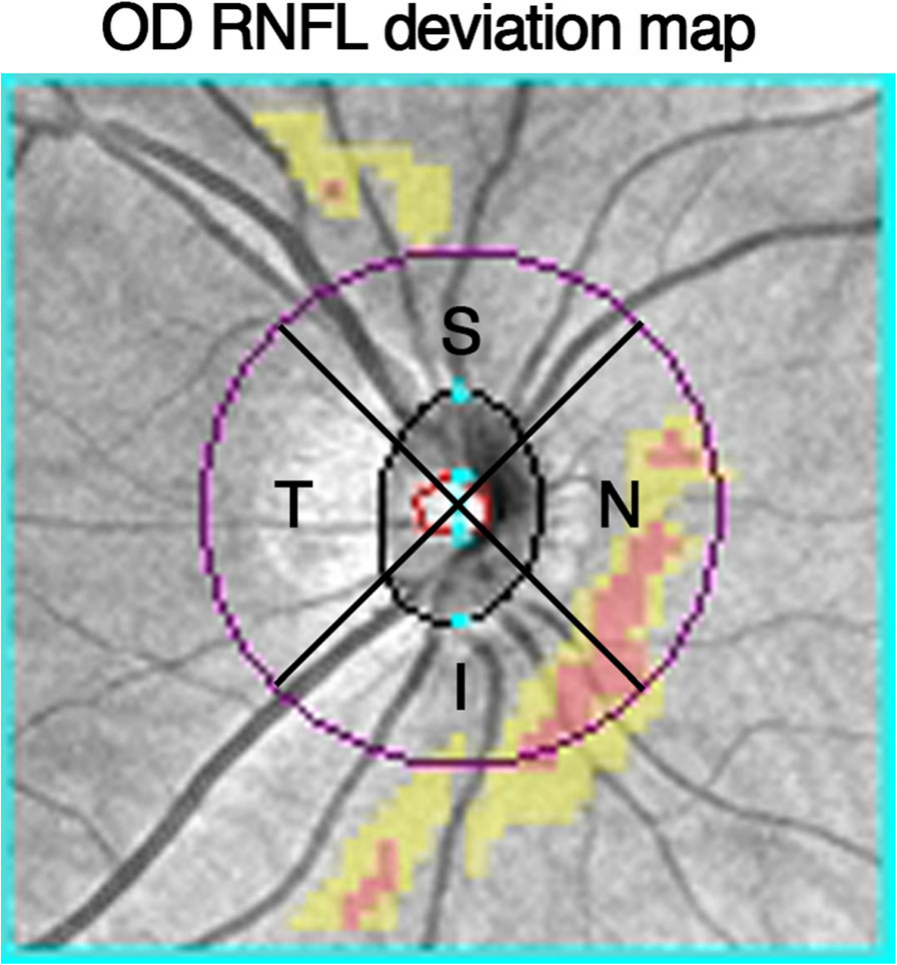
Retinal nerve fiber layer deviation map of the right eyes based on OCT

**Fig. 4:**
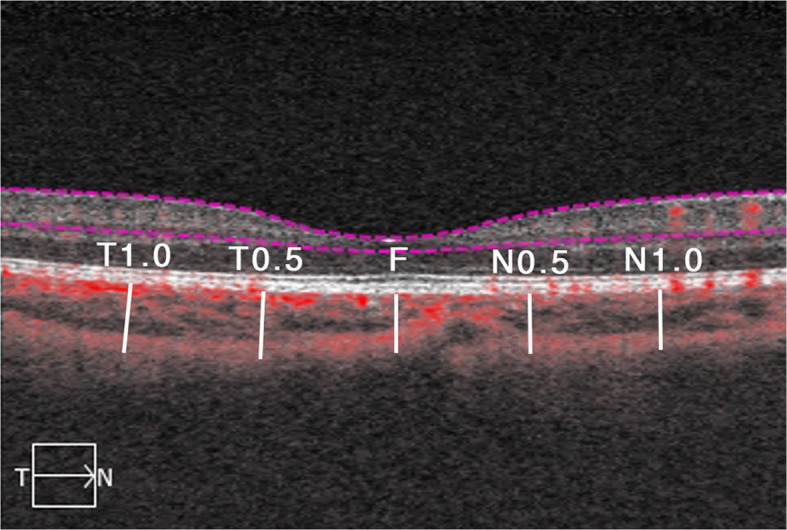
Choroidal thickness measurement of the fovea (F), 0.5 mm, and 1.0 mm from the fovea in nasal and temporal respectively (N0.5, N1.0, T0.5, T1.0)

MT, GCL, and RNFL were calculated automatically by OCT and shown in a map image. An MT map of nine zones from the internal limiting membrane (ILM) to the retinal pigment epithelium (RPE) was automatically calculated and recorded as M1-M9 (Fovea as M1, inner circle from superior to temporal as M2 to M5, outer circle from superior to temporal as M6 to M9) (Fig. 1). The GCL map of the six zones was recorded as G1–G6 (Fig. 2); the RNFL map of four zones was recorded as superior, nasal, temporal, and inferior (Fig. 3). CT was manually measured in the horizontal direction. The boundary of the choroid was defined from the hyper-reflective line of Bruch’s membrane to the line of the inner surface of the sclera. Fovea and points 0.5 mm, and 1.0 mm from the fovea in the nasal and temporal areas were measured (Fig. 4). Each point was measured three times for obtaining the mean value.

**Fig. 5:**
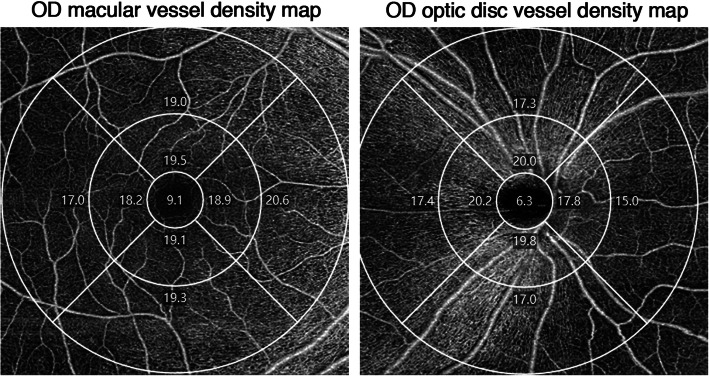
Vessel density map of the macular and optic disc of the right eyes based on OCTA

The vessel density map of the fovea and optic disk was a 6 mm diameter circle, divided into nine regions with three concentric circles. The inner one was 1.0 mm in diameter, middle one was 3 mm, and outer one was 6 mm. The circle was centred on the fovea and optic disc; the software automatically calculated values in each region of vessel density. Zones were recorded as A1–A9 and O1–O9, respectively, and the sequence was identical to the MT map (Fig. 5).

One skilled doctor obtained all the OCT scans. Images with signal strength higher than six were selected for analysis.

### Surgery process

Surgeries were performed using a VisuMax (Carl Zeiss Meditec) femtosecond laser platform by one experienced surgeon. Before surgery, 0.5% proparacaine hydrochloride (Alcon-couvreur N. V, Belgium) was used for anaesthesia. The suction time was 23 s for lenticule creation. The angle of the lenticule side cut was 90°. The cap depth was 120 μm with a diameter of 7.5 mm and a side cut angle of 120°. After the surgery, topical steroids (fluorometholone 0.1%; Santen Pharmaceutical Co., Ltd.) was used 6 times a day and reduced every 5–7 days over 30 days. Topical antibiotics (ofloxacin ophthalmic solution 0.5%; Santen Pharmaceutical Co., Ltd.) was used 4 times a day for 14 days. Artificial tears (sodium hyaluronate 0.1%, Santen Pharmaceutical Co., Ltd.) was used 4 times a day for at least 1 month.

### Statistical analysis

Statistical analysis was performed using SPSS 20.0 (IBM Corporation, Chicago, IL, USA). All values are expressed as mean ± SD. Analysis of paired Student’s t-test was used to assess the differences in CCT, ACD, LT, WTW, PD, MT, GCL, RNFL, CT, vessel density of macular and optic disk before and after surgery. Correlations between sphere and ΔACD and ΔLT were analysed with the Pearson correlation coefficient. Results were considered statistically significant when *p* < 0.05.

## Results

This study enrolled 61 eyes of 31 patients, including 21 females and 10 males. The mean age was 28.13 ± 5.84 years, ranging from 19 to 44 years. The mean sphere, astigmatism, and ablation depth are shown in Table 1.

**Table 1:** Characteristics of the subjects

Parameters	
Female/Male	21/10
Age (years)	27.81 ± 7.09
sphere (D)	−5.57 ± 1.67
astigmatism (D)	−0.62 ± 0.36
ablation depth (μm)	115.95 ± 24.42

AL, CCT, ACD, and PD decreased 1 day and 1 week after surgery. LT was thickened 1 day after surgery and became even thicker after 1 week. WTW did not change at either time point (Table 2).

**Table 2:** Ocular parameters measurement before and after surgery with Lenstar

	before surgery	1 day after surgery	p	1 week after surgery	p
AL (mm)	25.96 ± 1.01	25.86 ± 0.99	< 0.000	25.83 ± 1.00	< 0.000
CCT (μm)	540.23 ± 25.85	446.97 ± 30.48	< 0.000	439.25 ± 29.67	< 0.000
ACD (μm)	3.10 ± 0.29	3.04 ± 0.29	< 0.000	3.01 ± 0.30	< 0.000
LT (mm)	3.69 ± 0.33	3.71 ± 0.32	0.032	3.76 ± 0.33	< 0.000
WTW (mm)	11.95 ± 0.46	11.95 ± 0.51	0.955	11.95 ± 0.48	0.909
PD (mm)	5.22 ± 0.95	4.56 ± 0.80	< 0.000	4.69 ± 0.87	< 0.000

*AL* axial length, *CCT* central cornea thickness, *ACD* anterior chamber depth, *LT* lens thickness, *WTW* white to white, *PD* pupil diameter

**Fig. 6:**
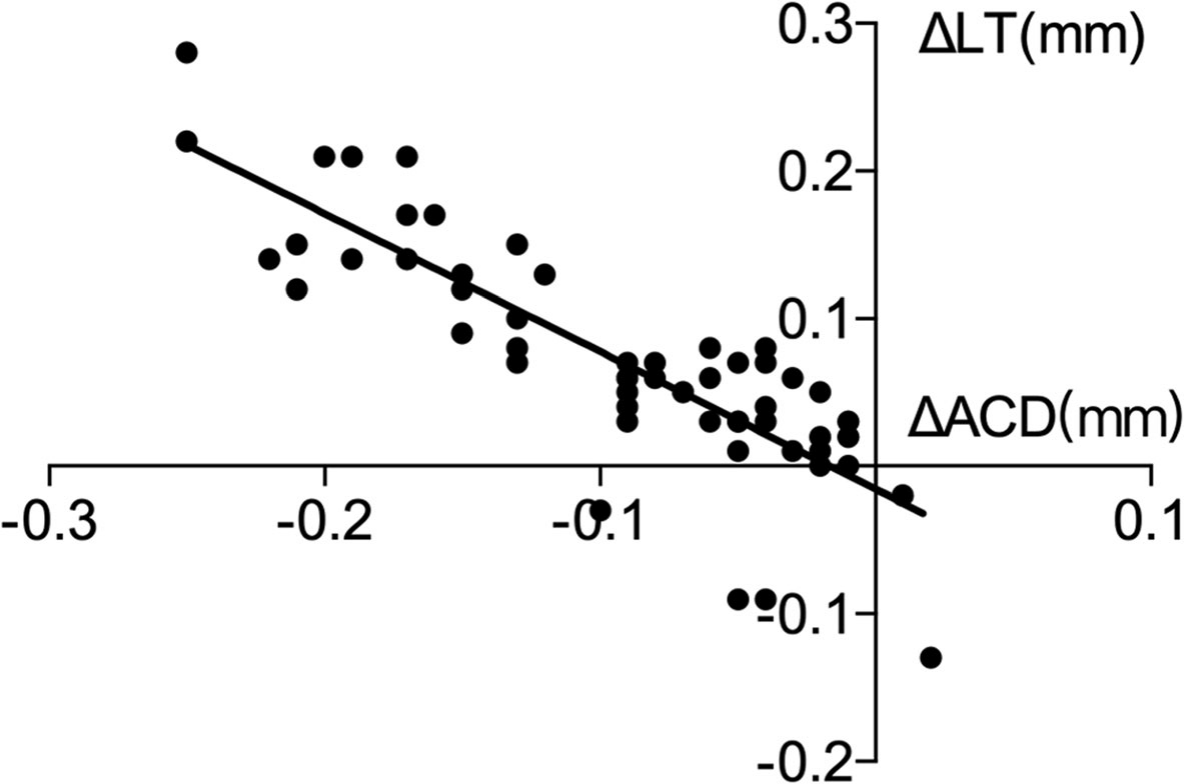
Correlation between ΔACD and ΔLT 1 week after surgery

**Fig. 7:**
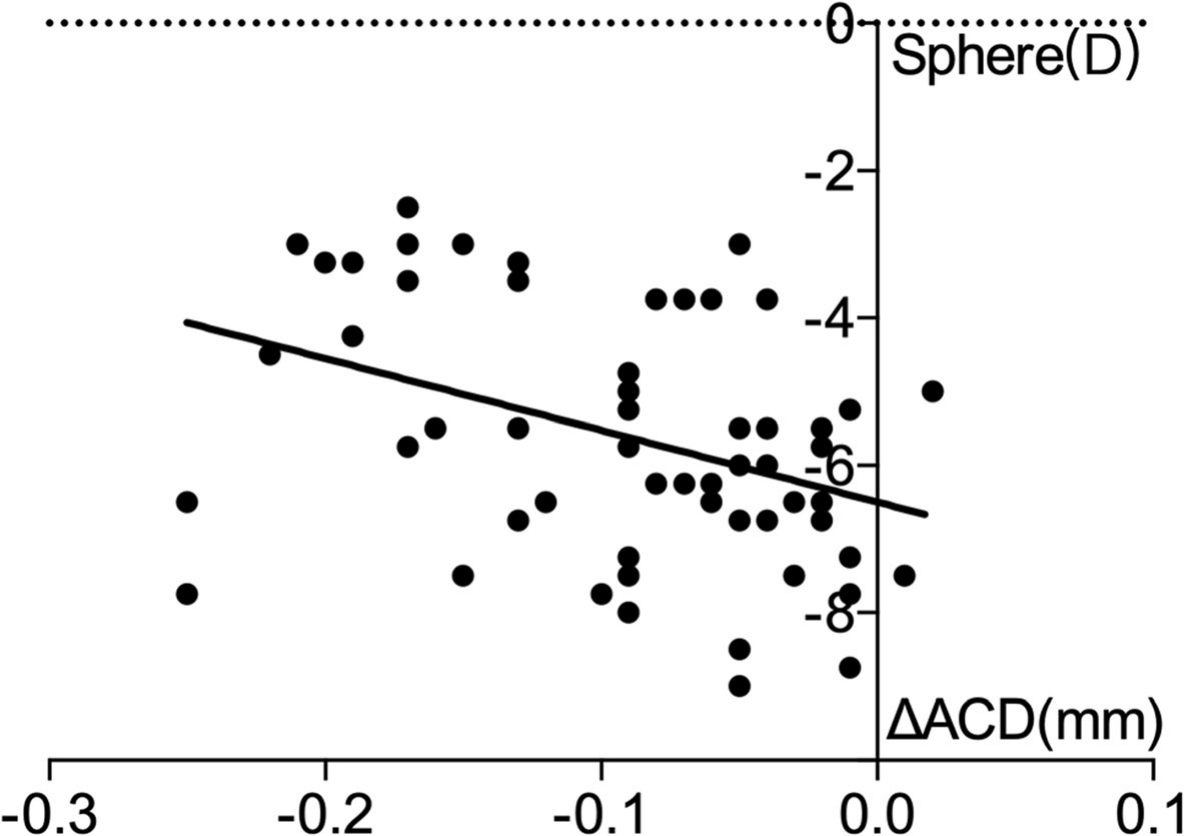
Correlation between ΔACD and sphere 1 week after surgery

**Fig. 8:**
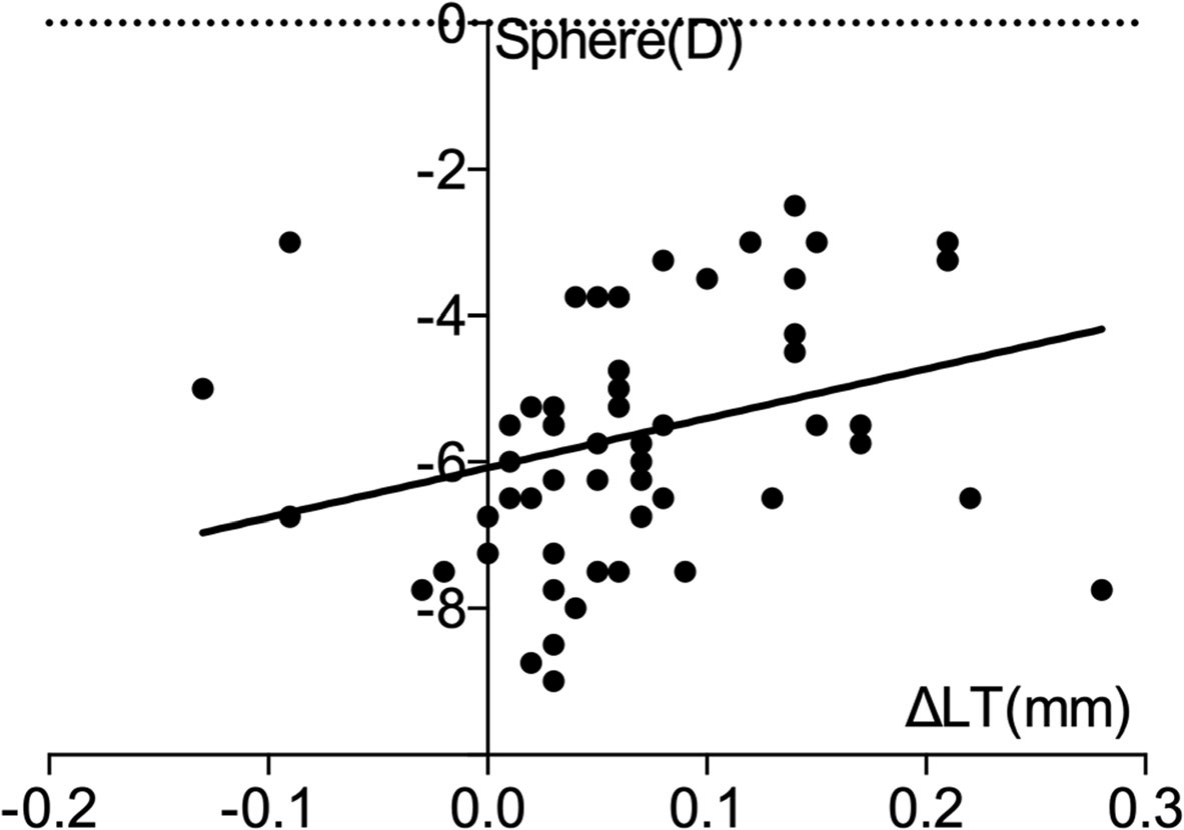
Correlation between ΔLT and sphere 1 week after surgery

ΔACD and ΔLT were not significantly correlated 1 day after surgery. ΔACD was negatively correlated with ΔLT and sphere 1 week after surgery (r = − 0.867, *p* < 0.000; r = − 0.398, *p* = 0.002). ΔLT was positively correlated with the sphere 1 week after surgery (r = 0.256, *p* = 0.048) (Figs. 6, 7 and 8).

One day after surgery, M1, M2, M3, and M5 were significantly reduced. One week after surgery, M1 and M5 were still thinner than the baseline. Moreover, M8 was thicker than the baseline 1 week after surgery (Table 3).

**Table 3:** Macular thickness (μm) before and after surgery with OCT

	before surgery	1 day after surgery	p	1 week after surgery	p
M1	248.88 ± 20.18	246.07 ± 19.41	< 0.000	246.26 ± 20.41	< 0.000
M2	319.16 ± 13.21	316.31 ± 16.17	0.009	318.21 ± 13.28	0.119
M3	319.36 ± 14.68	315.82 ± 16.95	0.003	317.80 ± 14.34	0.035
M4	309.69 ± 13.34	307.95 ± 13.88	0.105	309.49 ± 13.03	0.812
M5	303.62 ± 11.68	301.36 ± 12.56	0.001	302.49 ± 11.84	0.022
M6	278.57 ± 13.99	276.80 ± 12.94	0.190	278.26 ± 12.19	0.761
M7	294.72 ± 13.13	293.68 ± 12.13	0.276	293.80 ± 13.15	0.393
M8	257.31 ± 12.31	258.31 ± 11.43	0.273	259.34 ± 12.61	0.022
M9	254.64 ± 11.43	254.87 ± 10.08	0.740	255.34 ± 10.54	0.087

There were no significant changes observed in the GCL (Table 4).

**Table 4:** GCL thickness (μm) before and after surgery with OCT

	before surgery	1 day after surgery	p	1 week after surgery	p
G1	83.21 ± 5.39	82.83 ± 5.13	0.422	83.36 ± 4.74	0.696
G2	83.57 ± 4.74	83.67 ± 4.44	0.835	83.81 ± 4.43	0.520
G3	81.74 ± 6.11	81.72 ± 4.41	0.981	81.65 ± 4.70	0.859
G4	77.69 ± 5.43	78.47 ± 4.64	0.069	78.43 ± 5.80	0.152
G5	80.77 ± 4.75	81.21 ± 4.35	0.269	81.21 ± 4.41	0.077
G6	82.64 ± 6.30	81.89 ± 4.05	0.288	82.13 ± 4.07	0.392

The RNFL was thicker in the inferior segment 1 week after surgery (Table 5).

**Table 5:** RNFL thickness (μm) before and after surgery with OCT

	before surgery	1 day after surgery	p	1 week after surgery	p
superior	118.58 ± 17.42	118.90 ± 16.36	0.768	120.17 ± 15.56	0.118
nasal	62.41 ± 8.58	61.85 ± 7.43	0.687	61.56 ± 7.25	0.373
inferior	115.19 ± 12.38	116.66 ± 14.33	0.322	118.36 ± 14.15	< 0.000
temporal	96.25 ± 21.32	95.80 ± 20.85	0.595	94.80 ± 20.51	0.267

CT was detected thicker in the fovea 1 day after surgery and N1.0 1 week after surgery (Table 6).

**Table 6:** Choroidal thickness (μm) before and after surgery with OCT

	before surgery	1 day after surgery	p	1 week after surgery	p
F	274.80 ± 64.24	282.05 ± 65.28	0.041	281.18 ± 58.86	0.138
N0.5	254.57 ± 58.58	257.20 ± 62.80	0.360	258.74 ± 53.68	0.304
T0.5	262.34 ± 58.58	266.54 ± 51.79	0.143	271.65 ± 54.50	0.068
N1.0	239.34 ± 56.37	243.31 ± 57.48	0.147	249.15 ± 55.86	0.029
T1.0	266.32 ± 54.14	268.36 ± 49.17	0.365	268.13 ± 51.62	0.686

Macular vessel density decreased significantly 1 day after surgery, and most regions recovered after 1 week (Table 7).

**Table 7:** Macular vessel density (mm^−1^) before and after surgery with OCTA

	before surgery	1 day after surgery	p	1 week after surgery	p
A1	7.94 ± 3.02	5.00 ± 2.43	< 0.000	6.91 ± 2.53	0.003
A2	17.35 ± 2.06	14.17 ± 3.26	< 0.000	16.70 ± 1.88	0.055
A3	17.13 ± 2.23	14.48 ± 2.95	< 0.000	16.58 ± 2.09	0.118
A4	16.69 ± 2.43	13.97 ± 3.20	< 0.000	16.09 ± 2.45	0.112
A5	16.31 ± 2.66	13.47 ± 3.22	< 0.000	15.59 ± 2.32	0.047
A6	17.84 ± 1.59	15.92 ± 2.20	< 0.000	17.19 ± 1.57	0.012
A7	19.71 ± 1.20	18.44 ± 1.87	< 0.000	19.12 ± 2.25	0.053
A8	17.26 ± 2.11	15.06 ± 2.79	< 0.000	16.79 ± 1.93	0.140
A9	14.96 ± 3.06	13.17 ± 2.63	< 0.000	14.64 ± 2.33	0.463

Optic disc vessel density was reduced at the optic disc and peripapillary part (O5-O9) 1 day after surgery and recovered after 1 week (Table 8).

**Table 8:** Optic disc vessel density (mm^−1^) before and after surgery with OCTA

	before surgery	1 day after surgery	p	1 week after surgery	p
O1	6.63 ± 5.20	5.91 ± 4.75	0.001	6.38 ± 4.71	0.425
O2	19.14 ± 1.86	18.98 ± 1.99	0.550	19.35 ± 0.79	0.389
O3	17.90 ± 2.73	17.20 ± 3.63	0.047	18.08 ± 2.04	0.561
O4	18.95 ± 1.43	18.45 ± 2.54	0.099	18.93 ± 1.56	0.923
O5	18.36 ± 2.98	17.67 ± 3.35	0.014	17.83 ± 3.40	0.187
O6	17.84 ± 2.70	16.39 ± 3.20	< 0.000	17.60 ± 1.94	0.417
O7	15.19 ± 3.31	12.97 ± 3.72	< 0.000	14.49 ± 2.68	0.121
O8	17.26 ± 2.88	15.69 ± 3.27	0.001	16.97 ± 2.46	0.400
O9	17.77 ± 3.03	15.93 ± 3.75	0.001	17.45 ± 2.78	0.439

## Discussion

In this study, we measured several parameters and found that both the anterior segment and retina were affected by the surgery. Results from previous studies suggested that anterior segment changes would last for a long time, while posterior segment changes are only observed for a short time and then gets resolved. Prior studies have reported that PCE and ACD decreased after surgery and even several years later [6, 7], and this change was more significant in younger patients [12]. It has also been reported that changes in elevation correlated with residual bed thickness [13]. In this study, the ΔACD negatively correlated with sphere, this suggested that severe myopia was with more ablation depth and less residual bed thickness, leading to decreased cornea biomechanics and ACD. Besides, a negative correlation between ΔACD and ΔLT affirmed that thickened LT also attributed to the reduced ACD.

AL shortened by approximately 0.1 mm after surgery due to the ablation part. Corneas were usually oedematous after SMILE so that CCT was thinner 1 week than 1 day after surgery, and another article had the same result [14].

In a previous study regarding the treatment of presbyopia using a femtosecond laser, they found that the crystalline lens moved axially and laterally, and it seemed to be affected by suction [10]. The effect of suction usually lasts for a brief time. Our results showed that LT thickened in 1 day and were even thicker 1 week, so suction may not be the predominant factor. Other researchers found that LT increased after LASIK with four different instruments, and the pupil was dilated with 0.5% tropicamide before each measurement. The authors believed that residual accommodation might contribute to the LT increase [15]. In our study, all patients had natural pupils and were accompanied with thicker LT, and smaller PD than preoperative. This may prove the hypothesis that accommodation is enhanced after refractive surgery. Another study found that the amplitude of accommodation (AA) significantly decreased postoperatively. In our study, a slightly positive correlation between ΔLT and sphere suggested that severe myopia had less AA, which may explain that some patients complained of accommodation hysteresis after surgery, especially highly myopic patients. The poor accommodative ability, slow accommodative responsiveness, and increased accommodation demand may attribute to these results [16].

There are few articles reporting retinal or choroidal changes after SMILE, but changes after LASIK have been studied extensively. In previous LASIK studies, MT was thickened [11], or total macular volume increased [17]. 1 day after surgery, all parameters returned to baseline [18]. In our study, MT decreased after surgery, but GCL was unchanged, which was similar to prior study [19]. With the reduced M1, M2, M3, and M5, M8 increased in 1 week, which is contradictory to prior study findings on LASIK. The reason for decrease in MT and determination of the thinner layer require more in-depth studies.

Twenty years ago, RNFL changes after LASIK attracted doctors’ interests, and different results were concluded. RNFL was found to change with scanning laser polarimetry (SLP) but unchanged with OCT [20]. This result was caused by corneal birefringence [21], but not the real RNFL changes. Other researchers believed that the RNFL did reduce, but only for a very short time after surgery and soon recovers. Suction during surgery and high IOP caused disorders of the optic nerve axoplasm and malnutrition of retinal ganglia cells [11]. Nevertheless, research in children revealed that MT was thicker 1 day after surgery, but RNFL remained unchanged [22]. Another study found that the RNFL was thicker 3 months after LASIK, especially in the inferior-temporal sector [23], which was similar to our results, but the possible mechanism is still not clear.

CT was observed thickened postoperatively [16], and there is research believing that CT was affected by ciliary muscle contraction, which may explain why LT thickened. In our study, the results were similar, but not every measure point was statistically significant.

Vessel density has been studied extensively in glaucoma and retinal diseases since OCTA emerged. Vessel density changes were assumed by the suction effects during surgery on the retinal microcirculation, and instantaneous changes in suction may cause ischaemia-reperfusion injury [11]. IOP elevation during surgery also caused a decrease in ocular blood flow [24]. In a study on healthy people by increasing IOP, researchers found that transient elevation of IOP altered optic nerve head topography [25]. Other articles reported different results in a condition of natural IOP elevation and found no meaningful clinical impact [26]. In this study, macular vessel density and peripapillary vessel density were reduced. The recovery of macular vessel density was lower than peripapillary. Different retinal structures and sensitivity may be attributed for this effect. Reduced superficial vessel density also diminished vessel infusion, which may lead to thinner MT. Besides changes in the retina itself, the optical media may also affect OCT scanning due to mild corneal oedema postoperatively. The signal strength of images captured postoperatively was generally lower than that before surgery. This may be one reason for the reduced vessel density.

During the observation period, none of the patients had severe complications, and all of them regained ideal visual acuity. However, 1 day after the surgery, some corneas were not as clear as preoperatively due to mild corneal oedema, and it was difficult to capture high-quality images of the retina. Due to the limitation of the OCTA soft version, only superficial vessel density was analysed. These were the shortcomings of the study, along with the requirement of a longer follow-up period.

## Conclusion

After SMILE, the anterior segment was the most affected, while the retina also underwent changes with regard to MT, macular vessel density, and peripapillary vessel density. This study can help doctors gain deep insights into changes after refractive surgeries.

## Data Availability

All the relevant data of this study are available from the corresponding author upon request.

## Abbreviations

PRK: Photorefractive keratectomy
LASIK: Laser in situ keratomileusis
LASEK: laser subepithelial keratomileusis
SMILE: Small incision lenticule extraction
PCE: Posterior corneal elevation
OCTA: Optical coherence tomography angiography
IOP: Intraocular pressure
AL: Axial length
CCT: Central corneal thickness
ACD: Anterior chamber depth
LT: Lens thickness
WTW: White to white
PD: Pupil diameter
MT: Macular thickness
GCL: Ganglion cell layer
RNFL: Retinal nerve fiber layer
CT: Choroidal thickness
EDI: Enhanced depth imaging

## References

[1] Trokel SL, Srinivasan R, Braren B (1983). Excimer laser surgery of the cornea. Am J Ophthalmol.

[2] Vestergaard A, Ivarsen AR, Asp S, Hjortdal JO (2012). Small-incision lenticule extraction for moderate to high myopia: predictability, safety, and patient satisfaction. J Cataract Refract Surg.

[3] Blum M, Lauer AS, Kunert KS, Sekundo W (2019). 10-Year Results of Small Incision Lenticule Extraction. J Refract Surg.

[4] Wang D, Liu M, Chen Y, Zhang X, Xu Y, Wang J, CH T, Liu Q (2014). Differences in the corneal biomechanical changes after SMILE and LASIK. J Refract Surg.

[5] Chen M, Yu M, Dai J (2016). Comparison of biomechanical effects of small incision lenticule extraction and laser-assisted subepithelial keratomileusis. Acta Ophthalmol.

[6] Yu M, Chen M, Dai J (2019). Comparison of the posterior corneal elevation and biomechanics after SMILE and LASEK for myopia: a short- and long-term observation. Graefe's Arch Clin Exp Ophthalmol.

[7] Zhao Y, Jian W, Chen Y, Knorz MC, Zhou X (2017). Three-Year Stability of Posterior Corneal Elevation After Small Incision Lenticule Extraction (SMILE) for Moderate and High Myopia. J Refract Surg.

[8] Al-Sheikh M, Phasukkijwatana N, Dolz-Marco R, Rahimi M, Iafe NA, Freund KB, Sadda SR, Sarraf D (2017). Quantitative OCT angiography of the retinal microvasculature and the Choriocapillaris in myopic eyes. Invest Ophthalmol Vis Sci.

[9] Li M, Yang Y, Jiang H, Gregori G, Roisman L, Zheng F, Ke B, Qu D, Wang J (2017). Retinal microvascular network and microcirculation assessments in high myopia. Am J Ophthalmol.

[10] Kunert KS, Blum M, Reich M, Dick M, Russmann C (2009). Effect of a suction device for femtosecond laser on anterior chamber depth and crystalline lens position measured by OCT. J Refract Surg.

[11] Zhang J, Zhou YH (2015). Effect of suction on macular thickness and retinal nerve fiber layer thickness during LASIK used femtosecond laser and Moria M2 microkeratome. Int J Ophthalmol.

[12] Nishimura R, Negishi K, Dogru M, Saiki M, Arai H, Toda I, Yamaguchi T, Tsubota K (2009). Effect of age on changes in anterior chamber depth and volume after laser in situ keratomileusis. J Cataract Refract Surg.

[13] Zhou X, Shang J, Qin B, Zhao Y, Zhou X. Two-year observation of posterior corneal elevations after small incision lenticule extraction (SMILE)for myopia higher than -10 dioptres. Br J Ophthalmol. 2020;104(1):142-8. 10.1136/bjophthalmol-2018-313498 PMC6922016 31036587

[14] Liu T, Dan T, Luo Y (2017). Small incision Lenticule extraction for correction of myopia and myopic astigmatism: first 24-hour outcomes. J Ophthalmol.

[15] Wang L, Guo HK, Zeng J, Jin HY (2012). Analysis of changes in crystalline lens thickness and its refractive power after laser in situ keratomileusis. Int J Ophthalmol.

[16] Li M, Cheng H, Yuan Y, Wang J, Chen Q, Me R, Ke B (2016). Change in choroidal thickness and the relationship with accommodation following myopic excimer laser surgery. Eye (Lond).

[17] Feng L, Burns SA, Shao L, Yang Y (2012). Retinal measurements using time domain OCT imaging before and after myopic Lasik. Ophthalmic Physiol Opt.

[18] Zhang J, Zhou Y, Zheng Y, Liu Q, Zhai C, Wang Y (2014). Effect of suction on macular and retinal nerve fiber layer thickness during femtosecond lenticule extraction and femtosecond laser-assisted laser in situ keratomileusis. J Cataract Refract Surg.

[19] Zivkovic M, Jaksic V, Giarmoukakis A, Grentzelos M, Zlatanovic M, Zlatanovic G, Miljkovic A, Jovanovic S, Stamenkovic M, Kymionis G (2017). The effect of LASIK procedure on Peripapillary retinal nerve Fiber layer and macular ganglion cell-inner Plexiform layer thickness in myopic eyes. Biomed Res Int.

[20] Gurses-Ozden R, Liebmann JM, Schuffner D, Buxton DF, Soloway BD, Ritch R (2001). Retinal nerve fiber layer thickness remains unchanged following laser-assisted in situ keratomileusis. Am J Ophthalmol.

[21] Choplin NT, Schallhorn SC, Sinai M, Tanzer D, Tidwell JL, Zhou Q (2005). Retinal nerve fiber layer measurements do not change after LASIK for high myopia as measured by scanning laser polarimetry with custom compensation. Ophthalmology.

[22] Zhao PF, Zhou YH, Zhang J, Wei WB (2017). Analysis of macular and retinal nerve Fiber layer thickness in children with refractory amblyopia after femtosecond laser-assisted laser in situ Keratomileusis: a retrospective study. Chin Med J.

[23] Katsanos A, Arranz-Marquez E, Canones R, Lauzirika G, Rodriguez-Perez I, Teus MA (2018). Retinal nerve fiber layer thickness after laser-assisted subepithelial keratomileusis and femtosecond LASIK: a prospective observational cohort study. Clin Ophthalmol.

[24] Chen M, Dai J, Gong L (2019). Changes in retinal vasculature and thickness after small incision Lenticule extraction with optical coherence tomography angiography. J Ophthalmol.

[25] Piette S, Liebmann JM, Ishikawa H, Gurses-Ozden R, Buxton D, Ritch R (2003). Acute conformational changes in the optic nerve head with rapid intraocular pressure elevation: implications for LASIK surgery. Ophthalmic Surg Lasers Imaging.

[26] Zhang Q, Jonas JB, Wang Q, Chan SY, Xu L, Wei WB, Wang YX (2018). Optical coherence tomography angiography vessel density changes after acute intraocular pressure elevation. Sci Rep.

